# Recent advances in the treatment of venous thromboembolism in the era of the direct oral anticoagulants

**DOI:** 10.12688/f1000research.11174.1

**Published:** 2017-06-23

**Authors:** Jeffrey I. Weitz, Iqbal H. Jaffer, James C. Fredenburgh

**Affiliations:** 1 Department of Medicine, McMaster University, Hamilton, Ontario, Canada; 2 Department of Surgery, McMaster University, Hamilton, Ontario, Canada; 3 Department of Biochemistry and Biomedical Sciences, McMaster University, Hamilton, Ontario, Canada; 4 Thrombosis and Atherosclerosis Research Institute, Hamilton, Ontario, Canada

**Keywords:** direct oral anticoagulants, DOACs, venous thromboembolism, VTE, anticoagulant

## Abstract

The direct oral anticoagulants (DOACs) have now supplanted vitamin K antagonists (VKAs) for the treatment of venous thromboembolism (VTE). The DOACs include dabigatran, which inhibits thrombin, and rivaroxaban, apixaban, and edoxaban, which inhibit factor Xa. The DOACs are as effective for the prevention of recurrence as conventional VTE treatment, consisting of a parenteral anticoagulant followed by a VKA, and are associated with less bleeding. Because of these properties and the convenience of fixed dosing without the need for routine coagulation monitoring, guidelines now recommend DOACs over VKAs for VTE treatment in patients without active cancer. This paper examines the increasing role of the DOACs for VTE treatment.

## Introduction

Venous thromboembolism (VTE), which includes deep vein thrombosis (DVT) and pulmonary embolism (PE), is a common condition that occurs for the first time in about 1 in 1,000 persons each year, and the incidence rises with age ^1, 2^. About two-thirds of patients with symptomatic VTE present with DVT, while the remainder manifest as PE ^3^. Up to 12% of patients with PE and 6% of those with DVT die within 30 days ^4^. Of those who survive, 2 to 4% of PE patients develop chronic thromboembolic pulmonary hypertension, which can be fatal, and from 20 to 50% of DVT patients develop post-thrombotic syndrome, a chronic disorder characterized by leg swelling and pain that can lead to venous ulcers in severe cases ^5, 6^. Therefore, VTE is a common disorder associated with significant morbidity and mortality.

Anticoagulation is the cornerstone of VTE treatment. The goals of therapy are to prevent thrombus extension or embolization, to prevent new thrombi from forming, and to reduce the risk of long-term complications. Conventional VTE treatment consists of a parenteral anticoagulant, usually low-molecular-weight heparin (LMWH), overlapped and followed by a vitamin K antagonist (VKA), such as warfarin. Although effective and safe, conventional therapy is problematic because LMWH requires daily subcutaneous injection, which is difficult for some patients, and warfarin requires frequent monitoring and dose adjustments to ensure that the international normalized ratio (INR) is therapeutic, which is cumbersome for patients and physicians and costly for healthcare systems.

The treatment of VTE has been revolutionized with the recent introduction of the direct oral anticoagulants (DOACs), which can be given in fixed doses without routine monitoring. Four DOACs are licensed for VTE treatment: dabigatran, which inhibits thrombin, and rivaroxaban, apixaban, and edoxaban, which inhibit factor Xa. Their approvals were based on phase 3 trials demonstrating that the DOACs were as effective as conventional therapy but led to less bleeding. In patients without active cancer, DOACs are now favored over VKAs in official guidelines for the treatment of VTE because they are similarly effective, are safer, and provide the ease of fixed dosing without having to monitor coagulation ^7^. Focusing on the evolving use of the DOACs, in this paper we will (a) discuss the results of the phase 3 trials, (b) categorize VTE patients based on whether or not they are DOAC candidates, (c) demonstrate how to choose amongst the DOACs, (d) provide licensed dosing information for the DOACs, (e) review the optimal treatment duration for VTE, (f) describe the periprocedural management of the DOACs in patients needing surgery or intervention, and (g) evaluate the management of DOAC-associated bleeding.

## DOACs for the treatment of VTE

The DOACs were compared with conventional anticoagulation therapy in 27,023 patients with acute VTE in six trials: RE-COVER and RE-COVER II (Efficacy and Safety of Dabigatran Compared to Warfarin for 6-month Treatment of Acute Symptomatic Venous Thromboembolism) with dabigatran ^8, 9^, EINSTEIN DVT (Oral Direct Factor Xa Inhibitor Rivaroxaban in Patients with Acute Symptomatic Deep-Vein Thrombosis without Symptomatic Pulmonary Embolism) and PE (Oral Rivaroxaban for the Treatment of Symptomatic Pulmonary Embolism) with rivaroxaban ^10, 11^, AMPLIFY (Apixaban for the Initial Management of Pulmonary Embolism and Deep-Vein Thrombosis as First-line Therapy) with apixaban ^12^, and HOKUSAI VTE (Edoxaban versus Warfarin for the Treatment of Symptomatic Venous Thromboembolism) with edoxaban ^13^. The primary efficacy endpoint in these trials was recurrent VTE or VTE-related death, while the primary safety outcome was either major bleeding or the composite of major and clinically relevant non-major bleeding. In a pooled analysis ^14^, rates of recurrent VTE and VTE-related death were 2.0% with DOACs and 2.2% with conventional therapy (relative risk [RR] 0.90, 95% confidence interval [CI] 0.77–1.06). Compared with VKAs, the DOACs were associated with a 39% reduction in the risk of major bleeding (RR 0.61, 95% CI 0.45–0.83), a 63% reduction in intracranial bleeding (RR 0.37, 95% CI 0.21–0.68), and a 64% reduction in fatal bleeding (RR 0.36, 95% CI 0.15–0.84). In addition, clinically relevant non-major bleeding was reduced by 27% with the DOACs compared with VKAs (RR 0.73, 95% CI 0.58–0.93). Therefore, the DOACs demonstrate non-inferior efficacy compared with well-managed VKA therapy but are associated with significantly less bleeding ^14^.

Whereas dabigatran and edoxaban were started after a minimum 5-day course of parenteral anticoagulant therapy ^8, 9, 13^, rivaroxaban and apixaban were administered in all-oral regimens starting with higher doses for 21 days and 7 days, respectively ^11– 12^. When used in this all-oral fashion, both agents were non-inferior to conventional therapy and were associated with significantly less major bleeding. Therefore, the DOACs simplify VTE treatment and facilitate out-of-hospital management of most patients with DVT and many with PE, thereby reducing healthcare costs. With these advantages, it is not surprising that clinical guidelines now endorse DOACs as first-line VTE treatment ^7^.

Rivaroxaban, apixaban, and dabigatran were compared with placebo for extended treatment in VTE patients who completed at least 6 months of anticoagulation therapy in the EINSTEIN-extension (Once-daily Oral Rivaroxaban versus Placebo in the Long-term Prevention of Recurrent Symptomatic Venous Thromboembolism) ^11^, AMPLIFY-EXT (Apixaban after the Initial Management of Pulmonary Embolism and Deep Vein Thrombosis with First-line Therapy-extended Treatment) ^15^, and RE-SONATE (Twice-daily Oral Direct Thrombin Inhibitor Dabigatran Etexilate in the Long-term Prevention of Recurrent Symptomatic Venous Thromboembolism) ^16^ trials, respectively. In addition, dabigatran was compared with warfarin for extended therapy in the RE-MEDY trial (Dabigatran or Warfarin for Extended Maintenance Therapy of Venous Thromboembolism) ^16^, and rivaroxaban was compared with aspirin in the EINSTEIN CHOICE trial (Reduced-dose Rivaroxaban in the Long-term Prevention of Recurrent Symptomatic Venous Thromboembolism) ^17– 19^.

In the RE-MEDY study ^16^, dabigatran was non-inferior to warfarin for extended VTE treatment (hazard ratio [HR] 1.44, 95% CI 0.78–2.64) but was associated with a 46% reduction in major or clinically relevant non-major bleeding (HR 0.54, 95% CI 0.41–0.71). Pooled analyses of the three placebo-controlled trials revealed a significant reduction in the rate of recurrent VTE and VTE-related mortality with the DOACs but an increased rate of major and clinically relevant non-major bleeding ^17, 20^.

The AMPLIFY-EXT trial compared two dosing regimens of apixaban (2.5 mg and 5 mg twice daily) with placebo to identify the dose providing the best balance of efficacy and safety ^15^. The risks of recurrent VTE were similar with the lower- and higher-dose apixaban regimens (RR 0.97, 95% CI 0.46–2.02), and neither regimen was associated with a significant increase in major bleeding compared with placebo, but there was a trend for less non-major bleeding with the lower dose (RR 0.74, 95% CI 0.46–1.22).

Compared with placebo for extended VTE treatment, aspirin reduced the rate of recurrence by about 32% without a significant increase in major bleeding ^18, 21^. Based on this finding, guidelines now suggest aspirin for extended VTE treatment in patients who elect to stop anticoagulant therapy ^7^. The results of the EINSTEIN CHOICE trial challenge this suggestion ^19^. This trial compared two doses of rivaroxaban (20 mg and 10 mg once daily) with aspirin to identify the optimal dose of rivaroxaban for extended VTE treatment and to determine whether rivaroxaban is superior to aspirin for this purpose ^18^. The rates of recurrent VTE with the 20 mg and 10 mg rivaroxaban regimens were 1.5% and 1.2%, respectively, as compared with 4.4% in the aspirin group (HR 20 mg rivaroxaban versus aspirin 0.34, 95% CI 0.20–0.59 and HR 10 mg rivaroxaban versus aspirin 0.26, 95% CI 0.14–0.47; P<0.001 for both comparisons). Rates of major bleeding were 0.5% in the 20 mg rivaroxaban group, 0.4% in the 10 mg rivaroxaban group, and 0.3% in the aspirin group, and the rates of clinically relevant non-major bleeding also were similar (2.7%, 2.0%, and 1.8%, respectively) ^19^. Therefore, both dose regimens of rivaroxaban are superior to aspirin for the prevention of recurrent VTE and are associated with similar rates of bleeding. These results suggest that there is little role for aspirin for extended VTE treatment except for those who cannot afford rivaroxaban or have contraindications to its use.

## Choosing the right anticoagulant for the right patient

When faced with a patient with acute VTE, the first question to ask is whether the patient is suitable for DOAC treatment. Patients requiring thrombolytic therapy for high-risk PE associated with hypotension are usually started on heparin or LMWH but can be switched to a DOAC when their condition stabilizes. DOACs should be avoided in patients with renal impairment (creatinine clearance <15 mL/minute for rivaroxaban, apixaban, and edoxaban and <30 mL/minute for dabigatran), in those with severe hepatic impairment associated with coagulopathy, in those younger than 18 years of age, or in women who are pregnant or breastfeeding ( Table 1). VKAs remain the treatment of choice for VTE patients with a creatinine clearance <15 mL/minute and for those with antiphospholipid syndrome associated with arterial thrombosis. Although the data with DOACs in patients with cancer-associated VTE are promising ^14^, few such patients were included in the randomized trials. Consequently, guidelines recommend LMWH as first-line therapy in patients with cancer-associated thrombosis ^7, 22^. However, ongoing trials are comparing DOACs with LMWH in such patients ^23^.

**Table 1.** Patients with venous thromboembolism who are not candidates for direct oral anticoagulants.

Planned thrombolysis or intervention
Severe renal impairment (creatinine clearance less than 15 mL/minute)
Hepatic impairment with coagulopathy
Pregnant or breastfeeding
Younger than 18 years of age
Antiphospholipid syndrome with history of arterial thrombosis

DOACs should probably not be used in those with a body weight over 120 kg because data on their efficacy in such patients are lacking ^24^. Patients who cannot afford DOACs should receive conventional anticoagulant treatment because VKAs are less expensive. Finally, if compliance is a concern, or if the patient is taking multiple medications that may interact with the DOACs (including strong inhibitors of P-glycoprotein such as quinidine, verapamil, or dronedarone, or potent inducers or inhibitors of both P-glycoprotein and cytochrome P450 A34 isoenzymes such as carbamazepine, phenytoin, rifampin, St John’s wort, itraconazole, or ketoconazole), VKAs may be a better choice because INR monitoring will ensure therapeutic dosing.

In patients already taking VKAs and whose INR is erratic, VTE treatment should be replaced with a DOAC. This can also be considered in those for whom INR testing and dose adjustment is onerous, such as those with limited mobility. For long-term treatment, there is likely to be a lower risk of bleeding with the use of DOACs than with VKAs, particularly if the doses of apixaban or rivaroxaban are reduced to 2.5 mg twice daily and 10 mg once daily, respectively, after 6 months or more of full-dose treatment.

## Choosing amongst the DOACs

In VTE patients eligible for DOACs, there is no evidence to recommend one agent over another because head-to-head comparisons are lacking. Nonetheless, guidance can be provided ( Table 2). Creatinine clearance rates provide an essential metric in the decision-making process. For patients with creatinine clearance rates between 15 and 30 mL/minute, dabigatran should be avoided and an oral factor Xa inhibitor is suggested because they exhibit a lower dependence on renal excretion. To streamline transitions of care, rivaroxaban and apixaban should be considered because they have been evaluated in all-oral regimens, an approach that facilitates transitions from the clinic or the emergency department to home. Choice between the two depends on the ability to switch from the higher initial dose to the maintenance dose at 3 weeks or 1 week, respectively, and subsequently on patient preference for once- or twice-daily dosing regimens. In contrast, dabigatran and edoxaban were not evaluated as all-oral regimens and should be prescribed only after patients have completed a minimum 5-day course of treatment with LMWH or heparin. For patients over the age of 75 years with moderate renal impairment (creatinine clearance between 15 and 50 mL/minute) and low body weight, oral factor Xa inhibitors may be good choices because their benefit-to-risk profiles in such patients are superior to those of conventional therapy ^12, 25^.

**Table 2.** Choosing amongst the direct oral anticoagulants.

Characteristics	Drug Choice	Rationale
**CrCl 15–30 mL/minute**	Rivaroxaban, apixaban, or edoxaban	Less affected by renal impairment than dabigatran
**All-oral therapy**	Rivaroxaban or apixaban	Dabigatran and edoxaban require heparin bridging
**Dyspepsia or upper GI** **complaints**	Rivaroxaban, apixaban, or edoxaban	Dyspepsia with dabigatran in up to 10% of patients
**Recent GI bleed**	Apixaban or low-dose edoxaban	More GI bleeding with rivaroxaban and high-dose dabigatran or edoxaban than with warfarin
**Significant CAD**	Rivaroxaban, apixaban, or edoxaban	Possible small MI signal with dabigatran
**Poor compliance with** **twice-daily dosing**	Rivaroxaban or edoxaban	Only agents given once-daily

CAD, coronary artery disease; CrCl, creatinine clearance; GI, gastrointestinal; MI, myocardial infarction

It may be prudent to avoid dabigatran in patients with coronary artery disease because even though the rate of acute coronary syndrome with a 6-month course of dabigatran was similar to that with warfarin in RE-COVER and RE-COVER II (0.3% and 0.2%, respectively) ^8, 9^, the rate of acute coronary syndrome was higher with dabigatran than with warfarin in the RE-MEDY trial (0.9% and 0.2%, respectively; P=0.02), which compared them for extended VTE treatment for over a year ^16^. The rate of myocardial infarction also was higher with dabigatran than with warfarin in the RE-LY trial (Randomized Evaluation of Long-term Anticoagulation with Dabigatran Etexilate) ^26^. Although on re-analysis the difference was not statistically significant ^27^, meta-analyses suggest that the risk of myocardial infarction is higher with dabigatran than it is with warfarin ^28^. It is likely that the decreased bleeding with dabigatran compared with warfarin observed in RE-CORD, RE-CORD II, and RE-MEDY outweighs any small increase in acute coronary events; however, the oral factor Xa inhibitors offer the same safety advantage over warfarin and, in contrast to dabigatran, have not been associated with an increased risk of acute coronary syndrome. Therefore, with the uncertainty surrounding the increased risk of acute coronary syndrome with dabigatran compared with warfarin, an oral factor Xa inhibitor may be a better choice in patients with coronary artery disease.

Dabigatran also may not be the best choice for patients with upper gastrointestinal complaints because dyspepsia occurs in up to 10% of cases, although this tends to subside over time and often resolves when the drug is taken with food.

Although the risk of gastrointestinal bleeding was higher with the full-dose DOAC regimens than with warfarin in the phase 3 trials in patients with atrial fibrillation ^29^, this does not appear to be the case in VTE patients, probably because they are younger than those with atrial fibrillation, take fewer medications, and have fewer comorbidities. Thus, in a pooled analysis of the phase 3 VTE treatment trials, there was a non-significant trend for less bleeding with the DOACs than with VKAs (RR 0.77, 95% CI 0.49–1.21; p=0.11) ^30^.

The risk of bleeding with DOACs is increased with concomitant use of antiplatelet agents, such as aspirin and non-steroidal anti-inflammatory drugs, and these agents should be avoided if possible. For patients who must use aspirin, the daily dose of aspirin should not exceed 100 mg.

## Choosing the right dose of DOACs

To maximize efficacy, it is critical that the DOACs be used in the right dose. Depending on the agent, regulators have provided clinicians with dosing recommendations defined by characteristics including advanced age, reduced renal function, low body weight, and concomitant administration of potent P-glycoprotein inhibitors, factors associated with increased drug exposure and increased bleeding risk ( Table 3). Despite clear dosing recommendations, however, observational data suggest that the lower doses of the DOACs are over prescribed, potentially compromising the efficacy of DOACs in clinical practice ^31^. Education is needed to reverse this trend.

**Table 3.** Licensed direct oral anticoagulant dosing regimens for the treatment of venous thromboembolism.

	Dabigatran	Rivaroxaban	Apixaban	Edoxaban
Initial	After LMWH for at least 5 days, 150 mg BID	15 mg BID for 21 days	10 mg BID for 7 days	After LMWH for at least 5 days, 60 mg OD
Renal Adjustment	110 mg BID if ≥ 80 years, moderate renal impairment, or at risk for bleeding	N/A	N/A	30 mg OD if CrCl 15–50 mL/minute, weight <60 kg, or potent P-gp inhibitors
Long Term	150 or 110 mg BID	20 mg OD	5 mg BID	60 or 30 mg OD
Extended	Same dose	20 or 10 mg OD	2.5 mg BID	Same dose

BID, twice daily; CrCl, creatinine clearance; LMWH, low molecular weight heparin; N/A, not applicable; OD, once daily; P-gp, P-glycoprotein

## Optimal duration of VTE treatment

Optimizing the duration of anticoagulant therapy for VTE is important to minimize the risk of bleeding. All VTE patients require a minimum of 3 months of anticoagulant treatment. For patients with VTE provoked by a transient and reversible risk factor such as surgery, 3 months of anticoagulation is usually sufficient ^7^. In contrast, patients with ongoing risk factors, such as active cancer, or those with unprovoked VTE are often given extended anticoagulation therapy because their risk of recurrence is high if treatment is stopped ^7^. Therefore, many VTE patients require long-term anticoagulation therapy.

## Periprocedural management in patients receiving DOACs

Patients receiving long-term anticoagulant therapy often require elective surgery or invasive procedures, and appropriate perioperative management is important. To reduce the risk of bleeding complications, DOACs should be withheld for at least 24 hours before procedures associated with a moderate risk of bleeding and for at least 48 hours before procedures associated with a high risk of bleeding or if spinal anesthesia is planned ^32^.

Assessment of the anticoagulant effect of the DOACs or quantification of plasma drug levels can help guide the timing of surgery ( Table 4). These assessments depend on knowing which DOAC the patient is taking, the timing of intake of the last dose, and the impact of renal function on the half-life of the drug. Unfortunately, drug-specific tests are not widely or rapidly available. Regulators and hospitals need to work together to address this gap.

**Table 4.** Assays to measure the anticoagulant activity or plasma levels of the direct oral anticoagulants.

	Dabigatran	Rivaroxaban	Apixaban	Edoxaban
**PT**	✗	✔	✗	✗
**aPTT**	✔	✗	✗	✗
**dTT**	✔	✗	✗	✗
**ECT**	✔	✗	✗	✗
**Anti-FXa** **Assays**	✗	✔	✔	✔

aPTT, activated partial thromboplastin time; dTT, diluted thrombin time; ECT, ecarin clotting time; FXa, factor Xa; PT, prothrombin time

## Bleeding management in patients receiving DOACs

Managing bleeding with DOACs is done in a comparable manner to that with VKAs. When assessing a bleeding event, one must first determine how severe it is (i.e. mild, moderate-to-severe, or life-threatening) and where it is occurring (critical or non-critical site). Local measures can typically be employed to manage mild bleeding (e.g. epistaxis), but, in the case of persistent bleeds, it may be necessary to postpone the patient’s subsequent dose or to suspend treatment temporarily ^33^. Because DOACs have short half-lives, discontinuing their use normally results in rapid normalization of coagulation tests, as long as renal function is normal. The decision to briefly or permanently halt anticoagulation should always be taken with a view to balance the risk of bleeding against the risk of thrombosis.

In patients with moderate-to-severe bleeding events, supportive therapy is the mainstay of management ^33^. Because of the short half-life of the DOACs, most cases of bleeding will resolve within 12 hours provided that renal function is not severely compromised. The DOAC should be temporarily stopped as should concomitant long-acting antiplatelet agents (e.g. clopidogrel, ticagrelor, or prasugrel) if possible. Renal function should be assessed by measuring the serum creatinine and calculating the creatinine clearance. The anticoagulant effects or plasma levels of the DOACs can be determined using commercially available and validated assays ^34^ to assess the contribution of the DOAC to the bleeding event.

**Figure 1.**
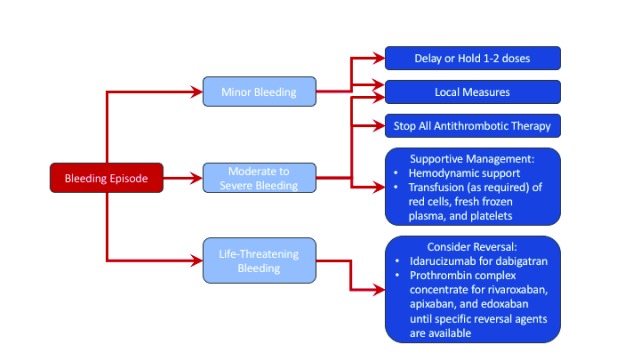
Management of direct oral anticoagulant-associated bleeding. With minor bleeding, local measures and delaying or holding 1–2 doses is sufficient. With moderate to severe bleeding, the direct oral anticoagulant should be held, and supportive therapy should be administered. For life-threatening bleeding, reversal should be undertaken.

Routine supportive measures include hemodynamic support with fluid replacement and administration of blood products, such as packed red blood cells, fresh-frozen plasma, and platelets if the patient has thrombocytopenia or if they were on long-acting antiplatelet agents ( Figure 1). The source of bleeding should be identified and, if possible, mechanical or surgical measures should be used to stop the bleeding; tranexamic acid can be considered. In the event of a DOAC overdose, gastric lavage and activated charcoal can be used within 2–4 hours of ingestion. An important aspect of bleeding management is to determine when reversal of the DOAC is indicated ^35^.

## Indications for DOAC reversal

The reversal of DOACs should be considered with life-threatening bleeding, such as intracranial hemorrhage, bleeding into a critical organ (e.g. intraocular bleeding) or a closed space (e.g. pericardial or retroperitoneal bleeding), ongoing bleeding despite supportive measures, and, particularly with dabigatran-associated bleeding, if there is associated acute kidney injury where a long delay in drug clearance is expected ( Table 5). Reversal should also be considered in patients who require urgent surgery or interventions that are associated with a high risk of bleeding and that cannot be delayed for at least 8 to 12 hours to allow the DOACs to clear from the circulation ^35^.

**Table 5.** Indications for reversal of direct oral anticoagulants.

Need for urgent surgery or intervention that cannot be delayed for at least 8 hours
Life-threatening bleeding (e.g. intracranial bleed)
Bleeding into a critical organ (e.g. intraocular bleed) or closed space (e.g. pericardial or retroperitoneal bleed)
Ongoing bleeding despite supportive measures
Expected long delay in restoration of hemostasis (e.g. over-anticoagulation with dabigatran in the setting of acute kidney injury)

## Reversal agents for the DOACs

Specific reversal agents include idarucizumab, which reverses only dabigatran, andexanet alfa, which reverses rivaroxaban, apixaban, edoxaban, and heparin, and ciraparantag, which reverses all of the DOACs and heparin ( Table 6). Of these, only idarucizumab is licensed and widely available; andexanet is under regulatory consideration, and ciraparantag has not yet been evaluated in patients. Until specific reversal agents for the oral factor Xa inhibitors are available, prothrombin complex concentrate should be considered.

**Table 6.** Features of specific reversal agents for the direct oral anticoagulants.

	Idarucizumab	Andexanet-alfa	Ciraparantag
**Structure**	Recombinant humanized Fab fragment	Recombinant human factor Xa variant	Synthetic small molecule
**Molecular mass (Da)**	48,000	39,000	513
**Synthesis**	Expressed in Chinese hamster ovary cells	Expressed in Chinese hamster ovary cells	Chemical synthesis
**Mechanism of action**	Binds dabigatran with high affinity	Competes with factor Xa for binding rivaroxaban, apixaban, or edoxaban	Binds direct oral anticoagulants via hydrogen bond formation
**Target**	Dabigatran	Rivaroxaban, apixaban, edoxaban, and heparin	Dabigatran, rivaroxaban, apixaban, edoxaban, and heparin
**Administration**	Intravenous bolus	Intravenous bolus followed by 2-hour infusion	Intravenous bolus
**Cost**	$3,500 per dose in United States	Unknown; likely to cost more than idarucizumab	Unknown; likely to cost less than idarucizumab and andexanet

## Conclusions and future directions

The DOACs are at least as effective, safer, and more convenient than VKAs and have streamlined VTE treatment. Post-marketing studies suggest that the favorable results of clinical trials can readily be translated into practice. Nonetheless, to optimize safety, there remains a need for selection of the appropriate patient, drug, and dose as well as careful follow up.

Although the DOACs represent a major advance in VTE treatment, gaps persist. For example, more information is needed about their utility in VTE patients with active cancer, their efficacy and safety in patients with a creatinine clearance between 15 and 30 mL/minute, and optimal dosing in obese and pediatric patients. Ongoing studies will help to address these gaps and enable DOAC use in a broader spectrum of VTE patients.
